# A bibliometric of publication trends in medical image segmentation: Quantitative and qualitative analysis

**DOI:** 10.1002/acm2.13394

**Published:** 2021-08-28

**Authors:** Bin Zhang, Bahbibi Rahmatullah, Shir Li Wang, Guangnan Zhang, Huan Wang, Nader Ale Ebrahim

**Affiliations:** ^1^ Data Intelligence and Knowledge Management, Faculty of Arts, Computing and Creative Industry Sultan Idris Education University (UPSI) Tanjong Malim Perak Malaysia; ^2^ School of Computer Science Baoji University of Arts and Sciences Baoji P. R. China; ^3^ Research and Technology Department Alzahra University Vanak Tehran Iran; ^4^ Office of the Deputy Vice‐Chancellor (Research & Innovation) University of Malaya Kuala Lumpur Malaysia

**Keywords:** bibliometric, image segmentation, medical image, publication trends, research productivity

## Abstract

**Purpose:**

Medical images are important in diagnosing disease and treatment planning. Computer algorithms that describe anatomical structures that highlight regions of interest and remove unnecessary information are collectively known as medical image segmentation algorithms. The quality of these algorithms will directly affect the performance of the following processing steps. There are many studies about the algorithms of medical image segmentation and their applications, but none involved a bibliometric of medical image segmentation.

**Methods:**

This bibliometric work investigated the academic publication trends in medical image segmentation technology. These data were collected from the Web of Science (WoS) Core Collection and the Scopus. In the quantitative analysis stage, important visual maps were produced to show publication trends from five different perspectives including annual publications, countries, top authors, publication sources, and keywords. In the qualitative analysis stage, the frequently used methods and research trends in the medical image segmentation field were analyzed from 49 publications with the top annual citation rates.

**Results:**

The analysis results showed that the number of publications had increased rapidly by year. The top related countries include the Chinese mainland, the United States, and India. Most of these publications were conference papers, besides there are also some top journals. The research hotspot in this field was deep learning‐based medical image segmentation algorithms based on keyword analysis. These publications were divided into three categories: reviews, segmentation algorithm publications, and other relevant publications. Among these three categories, segmentation algorithm publications occupied the vast majority, and deep learning neural network‐based algorithm was the research hotspots and frontiers.

**Conclusions:**

Through this bibliometric research work, the research hotspot in the medical image segmentation field is uncovered and can point to future research in the field. It can be expected that more researchers will focus their work on deep learning neural network‐based medical image segmentation.

## 1 INTRODUCTION

Medical images, including but not limited to magnetic resonance imaging (MRI), computed tomography (CT), X‐ray, and ultrasound, are key components of the diagnosis and treatment plan.^1^ However, the evaluation and segmentation results of medical images are often different from person to person, and sometimes even get error results.^2^, ^3^ With the increasing volume of medical images, it has become indispensable to use computers for processing and analyzing these data.^4^ In particular, medical image segmentation algorithms can assist medical experts to segment the region of interest in medical images automatically, which can be used for tissue volume, diagnosis, the study of anatomical structure, and computer integrated surgery.^5^, ^6^, ^7^ Image segmentation algorithms can highlight regions of interest and remove unnecessary information,^8^ while the quality of these algorithms will directly affect the performance of the following processing steps.^9^

Bibliometric refers to the interdisciplinary study of quantitative analysis of all knowledge carriers by means of mathematics and statistics.^10^ There are many bibliometric analysis works focused on different fields of research including toxicology and industrial health,^11^ chronic obstructive pulmonary disease,^12^ medical data mining research,^13^ drug delivery.^14^ At the same time, there are also some reviews on the medical image segmentation field such as 3D medical image segmentation,^15^ current methods in medical image segmentation,^1^ automated medical image segmentation techniques,^16^ medical image segmentation on graphics processing units (GPUs),^9^ medical image segmentation applied to the female pelvic cavity research,^17^ fetal ultrasound image segmentation,^18^ and genetic‐based medical image segmentation.^19^ However, some reviews on medical image segmentation only studied a special category of segmentation algorithm,^9^, ^17^, ^19^ and most of them had been published for a long time, so they cannot grasp the research trends and hotspots in this field. To our knowledge, there is no bibliometric analysis work in the medical image segmentation field published in recent years. However, there are some limitations to using quantitative analysis alone,^20^ and it needs to be combined with qualitative analysis to get more accurate and reliable analysis results.^21^ According to this, top publications were filtrated based on the annual citation rates for qualitative analysis.^22^ The total citation number and annual citation rates are important indicators of the academic influence of publications.^23^ The purpose of this work is to explore the research status and trends of the medical image segmentation field, enable researchers to grasp the development history and the current research hotspot of this field. To achieve this target, both bibliometric analysis and qualitative analysis have been used.

The structure of this article is as follows: In the first section, the background and purpose of this study are introduced. In the second section, the research methodology, the data sources, and bibliometric tools are described. In the third section, quantitative analysis has been implemented from five perspectives including annual publication, countries, authors, sources, and keywords. In the fourth section, qualitative analysis has been implemented to top annual citation rates publications. In the last section, the conclusion of this study is presented.

## 2 MATERIALS AND METHODS

Using bibliometric combined with qualitative analysis, the publication trends in the medical image segmentation field were evaluated. Two well‐known academic databases, the WoS Core Collection and Scopus, were used to collect relevant publications. The WoS database is an internationally recognized database that can reflect high‐level scientific research. It enjoys a good reputation in the world of science, technology, and education with “Science citation index Expanded” (SCIE), “Social Science Citation Index” (SSCI), and other citation index databases, besides journal citation reports (JCR) and essential science indicators (ESI). It is the world's largest academic database, covering over 15 000 scientific, technical, and medical journals. WoS core collection includes SCIE, SSCI, Arts and Humanities Citation Index (A&HCI), and Emerging Source Citation Index (ESCI). Scopus covers the world's most extensive scientific and medical publications in abstracts, references, and indexes, which collects publications from many famous journals.^24^

**FIGURE 1:**
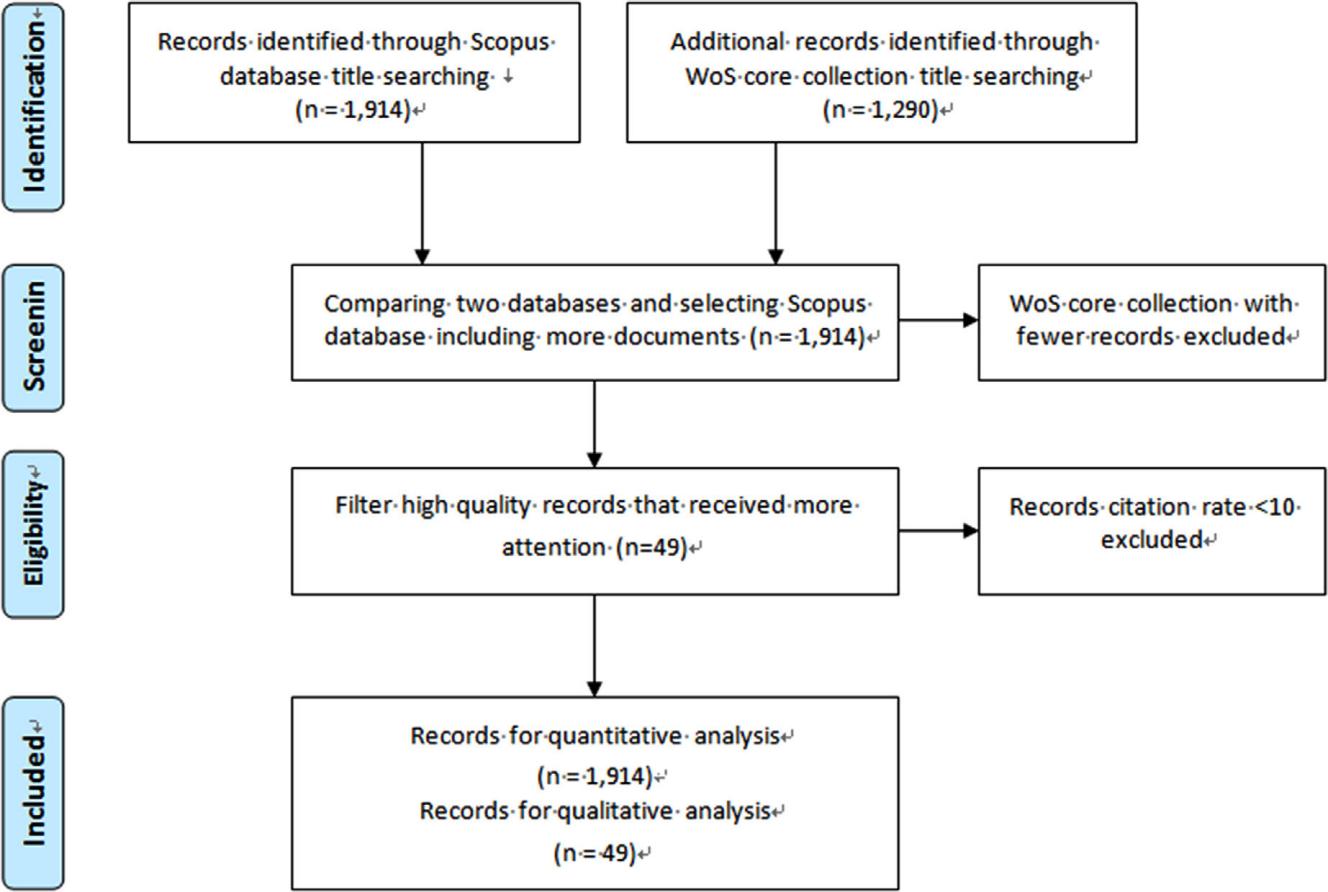
The data collection and filtering process for the bibliometric analysis of medical image segmentation

The data collection and filtering process are shown in Figure 1, as “Preferred Reporting Items for Systematic Reviews and Meta‐Analyses” (PRISMA)^25^ flow diagram. The query sentence “(ROI OR “region of interest” OR segmen*) AND (“medical imag*” OR DICOM)” was run on both databases, to retrieve publications with the keywords in their title, and both the queries were performed on January 11, 2021. The number of publications retrieved was 1290 records from the WoS core collection and 1914 records from the Scopus. Search on the WoS core collection retrieved fewer records than the same search on Scopus, and most of them were duplicated. In fact, about 95 percent of documents in most research areas are included in both the WoS and Scopus databases.^26^ Therefore, a bibliometric analysis was conducted for these 1914 records in Scopus from five quantitative perspectives including annual publications, countries, top authors, sources, and keywords. The analytical tool used for this analysis was bibliometrix package. Bibliometrix package is an R tool designed by two Italian scholars, Massimo Ariaa and Corrado Cuccurullo.^27^ By using this tool, bibliographic data that gets from well‐known academic databases, such as Scopus and WoS, can be analyzed quantitatively. Then the results of quantitative analysis of bibliometric are combined with qualitative analysis of the content of publications. Among the 1914 records, the first 49 publications whose annual citation rates were no less than 10 were filtered for qualitative analysis. Taxonomy was conducted to classify these publications into three categories: reviews, segmentation algorithm publications, and other relevant publications. The following section will provide a detailed analysis of the collected records.

## 3 QUANTITATIVE ANALYSIS

Up to January 11th, 2021, a total number of 1914 publications related to medical image segmentation have been collected. In this section, a quantitative analysis of these publications will be conducted from five perspectives: annual publications analysis, countries and languages analysis, top authors analysis, sources analysis, and keywords analysis.

### 3.1 Annual publications analysis

**FIGURE 2:**
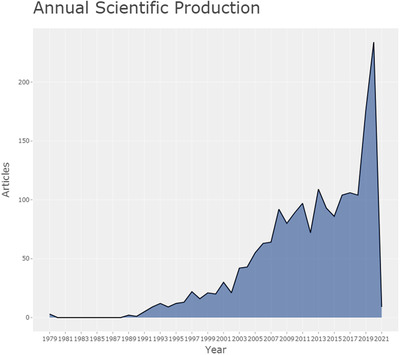
Annual scientific production of medical image segmentation research field as of January 11th, 2021

Figure 2 shows the annual scientific production on “medical image segmentation” as of January 11th, 2021. There were a total number of 1914 publications, including 1045 conference papers which accounted for 54.6%, 775 journal articles which accounted for 40.5%, 17 reviews, and a few other kinds of publications. Since the 1990s, the number of publications in this field has increased from 0 to 234 per year in 2020, showing a rapid growth trend of the annual publication.

The number of publications has grown at an average annual rate of 18.7% over the past 30 years. Stripping out the impact of the earlier years of a low number of publications, the average annual growth rate over the past decade has been 13.6%. Compared with all publications included in the Scopus database, the number of published articles has increased from 881 500 in 1990 to 2 712 413 in 2020, with an average annual growth rate of only 3.8%, and an average annual growth rate of 4.3% in the past decade. This indicates that the number of publications growth speed of the medical image segmentation research field is significantly higher than that in overall scientific research.

The citations of these 1914 publications are analyzed as a whole. These publications were cited a total number of 29 669 times, among which 27 752 times were cited by others, accounting for 93.5%. The number of self‐citation was 1917 times, accounting for 6.5%. The general view is that a high other‐citation rate indicates the high quality of the article. Therefore, the subsequent citation analysis in this article excludes self‐citation and only analyzes other citation data.

**FIGURE 3:**
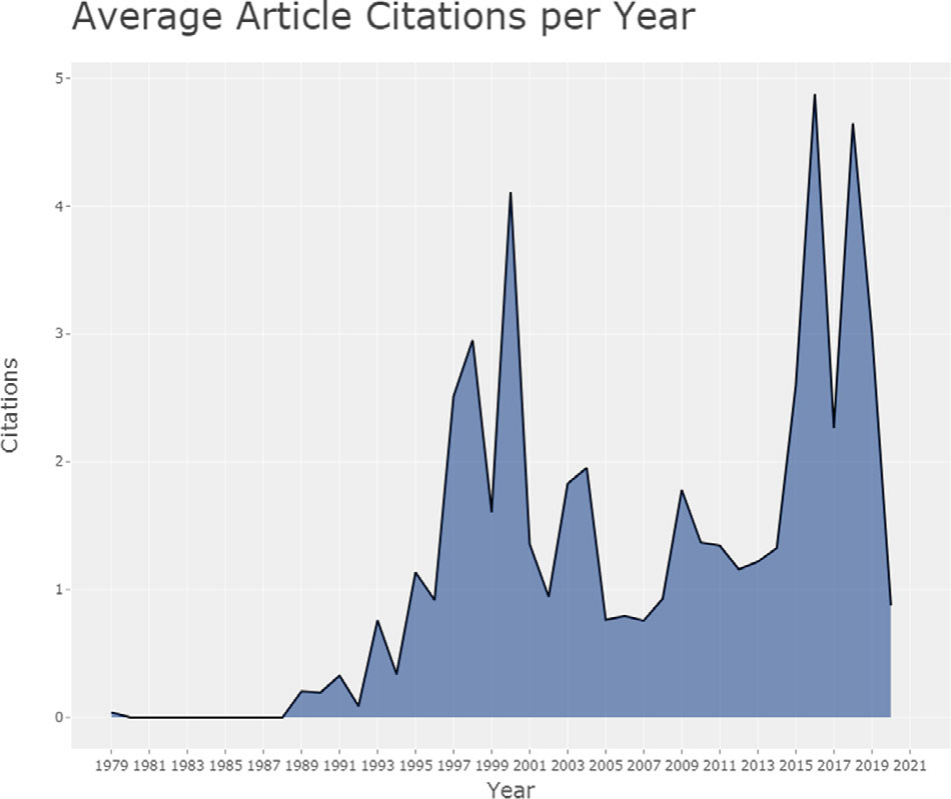
Average article citations per year of medical image segmentation research field as of January 11th, 2021

The average article citations per year are shown in Figure 3. Since the publications in the medical image segmentation field emerged in the 1990s, the average article citations per year have gone through three periods. In the 1990s, the average article citations per year increased gradually and reached the first peak of 4.1 in 2000. Then it went through a 15‐year trough until 2016, when the average article citations per year reached another peak and hold to now.

### 3.2 Countries and languages analysis

**FIGURE 4:**
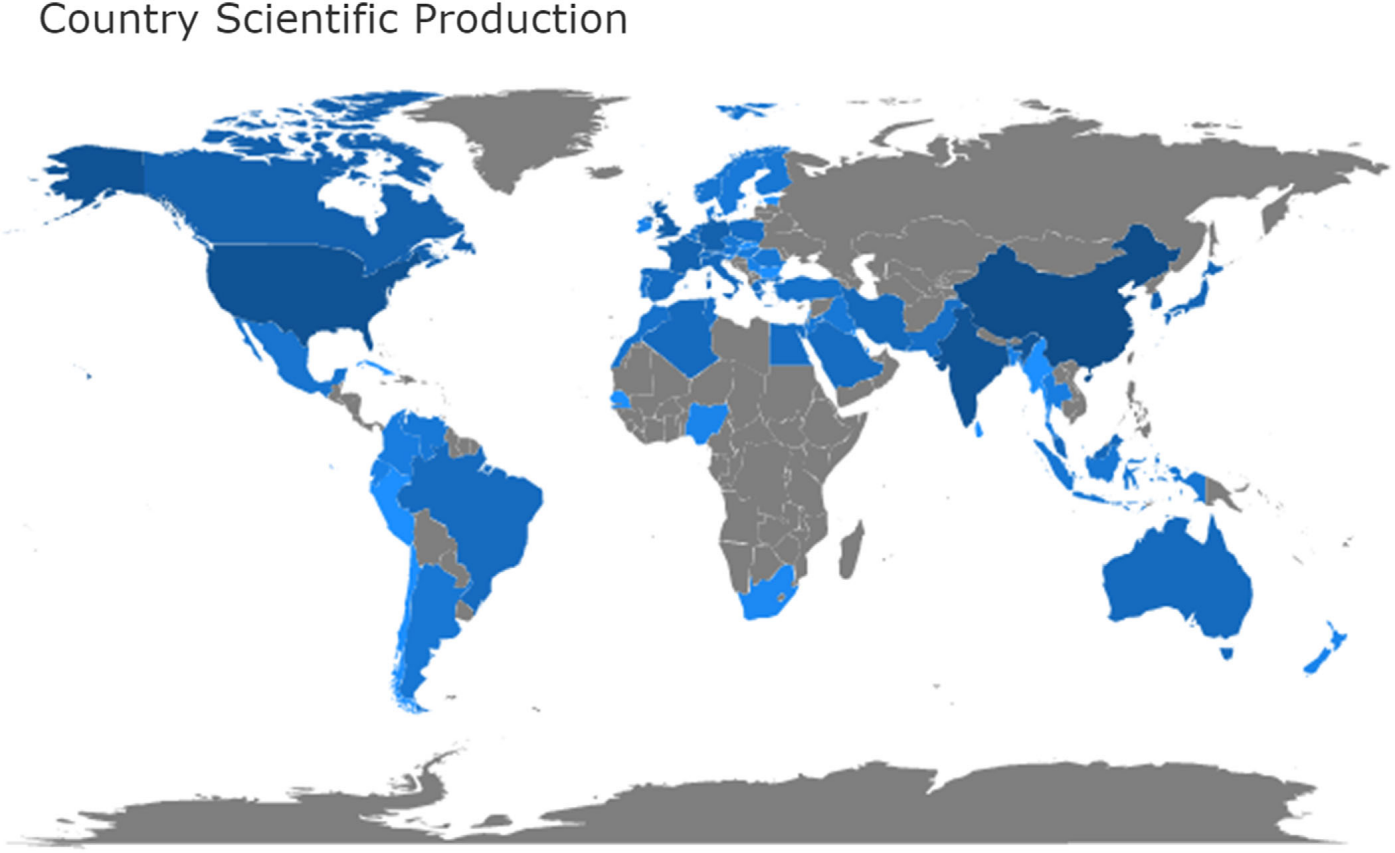
Country or region scientific production world map in the medical image segmentation field. (blue color: country or region with publications, grey color: country orregion without publications, the intensity: the publications’ number)

In our analysis set of 1914 publications, corresponding authors are distributed in 54 countries or regions, as shown in Figure 4. On the world map, the blue countries or regions means that there are corresponding authors from there, while the intensity of the blue is proportional to the number of publications in the country or region, and the deeper of the blue, the more articles were published. It is easy to see that the Chinese mainland (excluding Hong Kong, Macao, and Taiwan, the same below), the United States, and India have a darker color than any other country or region. In fact, these three countries also have the largest number of publications, accounting for 32.1, 16.4, and 8.3%, respectively. Besides these three countries, there are also some other countries whose publications account for more than 1% of the total including South Korea, the United Kingdom, Canada, Germany, France, Japan, Italy, Singapore, Malaysia, Iran, Poland, Algeria, Greece, and Spain. The major participants included nine western countries, seven Asian countries, and one African country.

**FIGURE 5:**
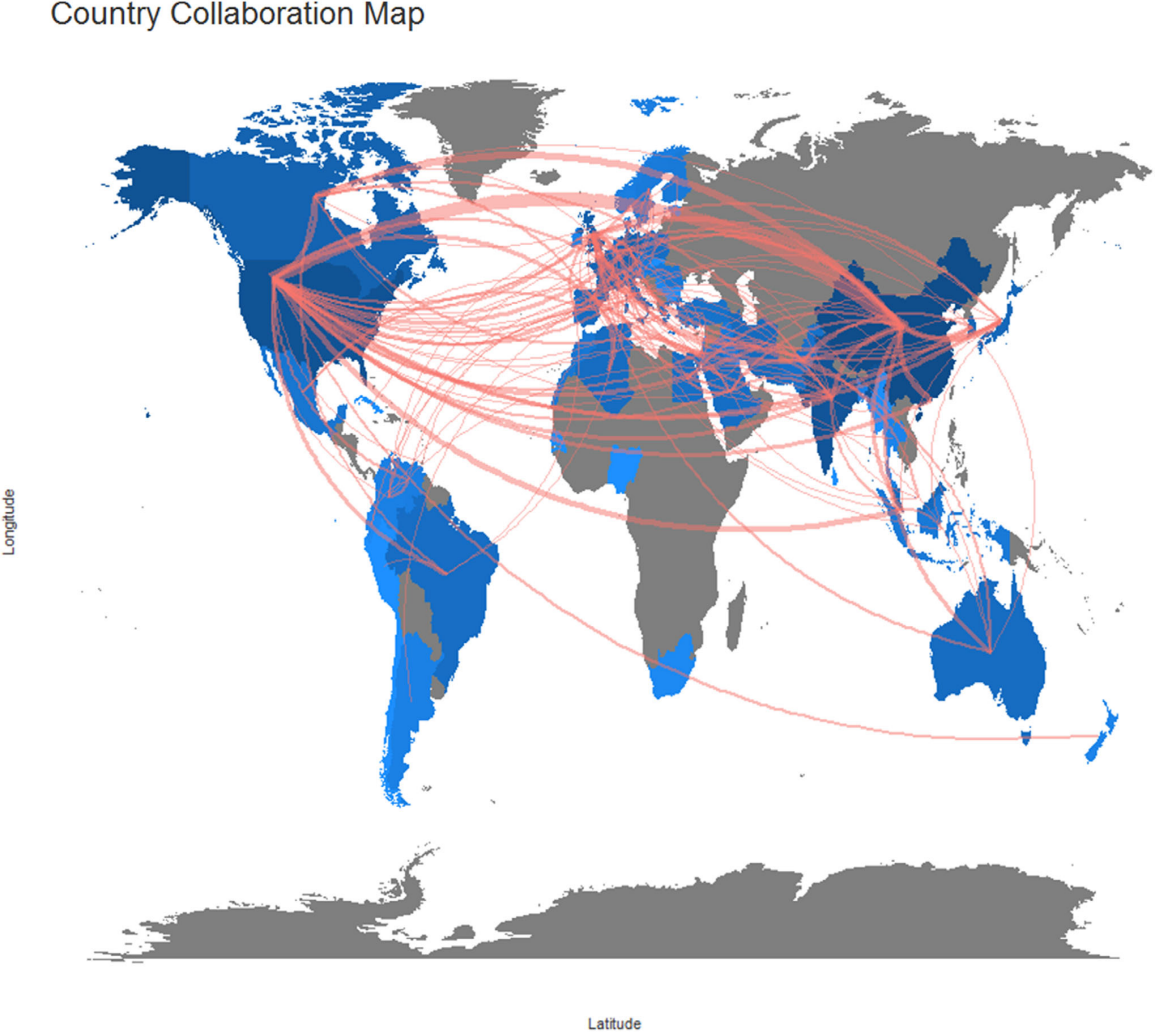
Country or region collaboration world map in the medical image segmentation field. (blue color:country or region with publications, grey color: country or region without publications, color intensity: the publications’ number, red lines’ thickness: Co‐published papers’ number)

Figure 5 shows the countries or regions collaboration map in the medical image segmentation field. If there is some collaboration between two countries or regions, they will be connected with red lines, and the thickness of the red line is proportional to the number of collaborations. As Figure 5 illustrates, the Chinese mainland and the United States seem to be the hub country of publications, because there are lots of red lines between the Chinese mainland, the United States, and other countries or regions. In particular, the collaboration between the United States and the Chinese mainland has 40 copublished papers, which occupies the maximum thickness.

**FIGURE 6:**
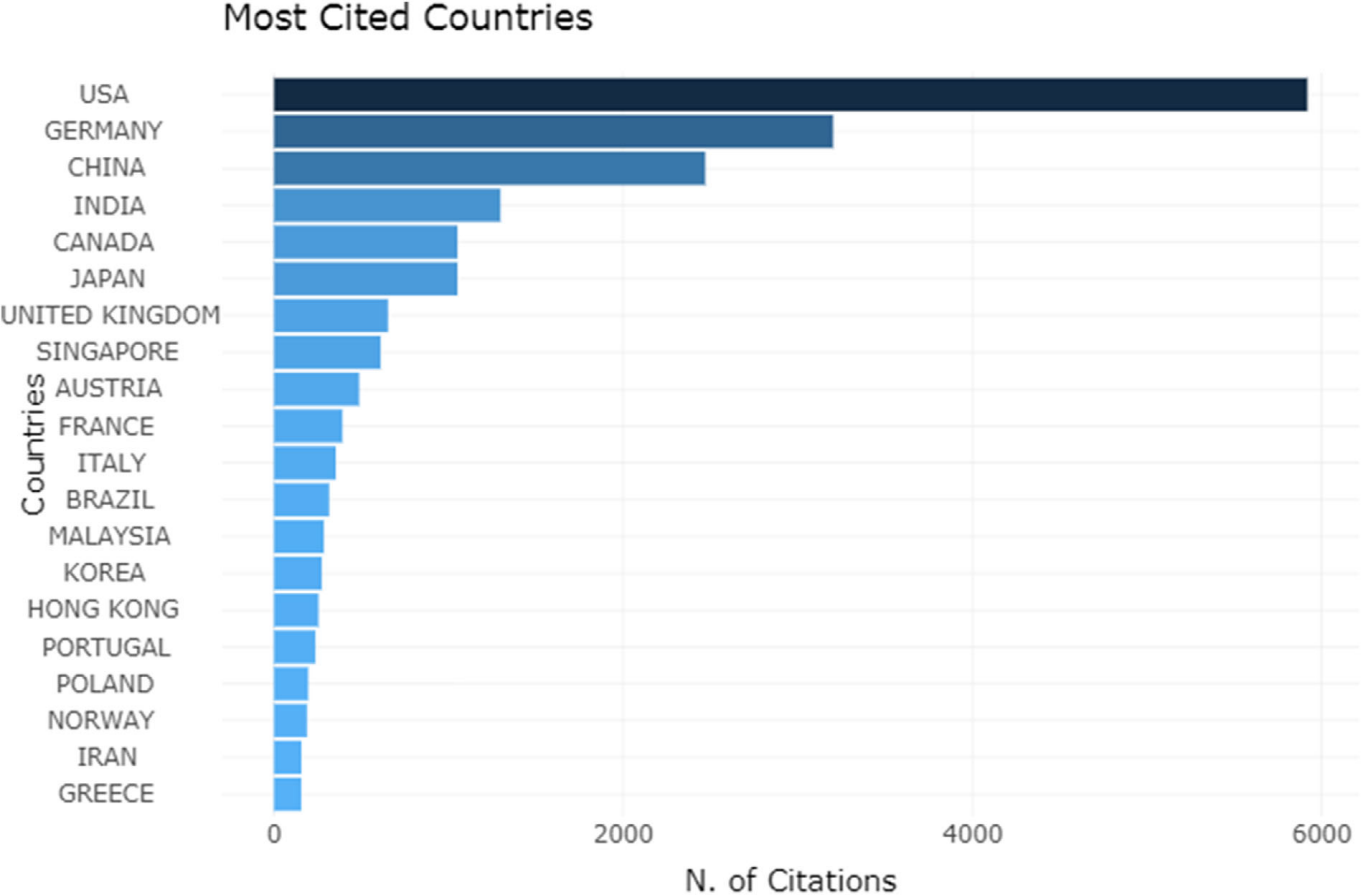
Top 20 most cite dcountries or regions and the number of citations

Figure 6 shows the top 20 most cited countries or regions. It is easy to see that the United States authors lead the way in citations. Although German authors did not publish as many articles as the Chinese mainland and India, their publications are cited more often than the Chinese mainland and India.

The publications from Austrian or German authors have more than 100 average article citations, which is much higher than any other country or region in terms of citation intensity. The average article citations of the major participating countries or regions are listed in Table 1. As the top three countries with the number of publications, the average article citations of the Chinese mainland, the United States, and India are not too high, among which the Chinese mainland only has 7.94 average article citations.

**TABLE 1:** Average article citations of the major participating countries or regions

Country or region	Average article citations
Austria	122.50
Germany	114.36
Japan	58.39
USA	37.22
Singapore	38.19
Canada	36.24
Hong Kong	28.33
United Kingdom	21.77
Italy	20.82
Malaysia	19.07
France	17.04
India	16.21
Poland	14.00
China	7.94

Table 2 lists the languages of publication in detail. In our analysis set of 1914 publications, most of the literature was written in English, accounting for 95%, and the second most widely used language is Chinese, but it accounts for only 4.3%. There are also a small number of publications in Turkish, Spanish, German, French, Japanese, Portuguese, and other languages.

**TABLE 2:** The language of publications and their number

Language	Number of Publication
English	1822
Chinese	82
Turkish	5
Spanish	3
German	2
French	1
Japanese	1
Portuguese	1

### 3.3 Authors analysis

**FIGURE 7:**
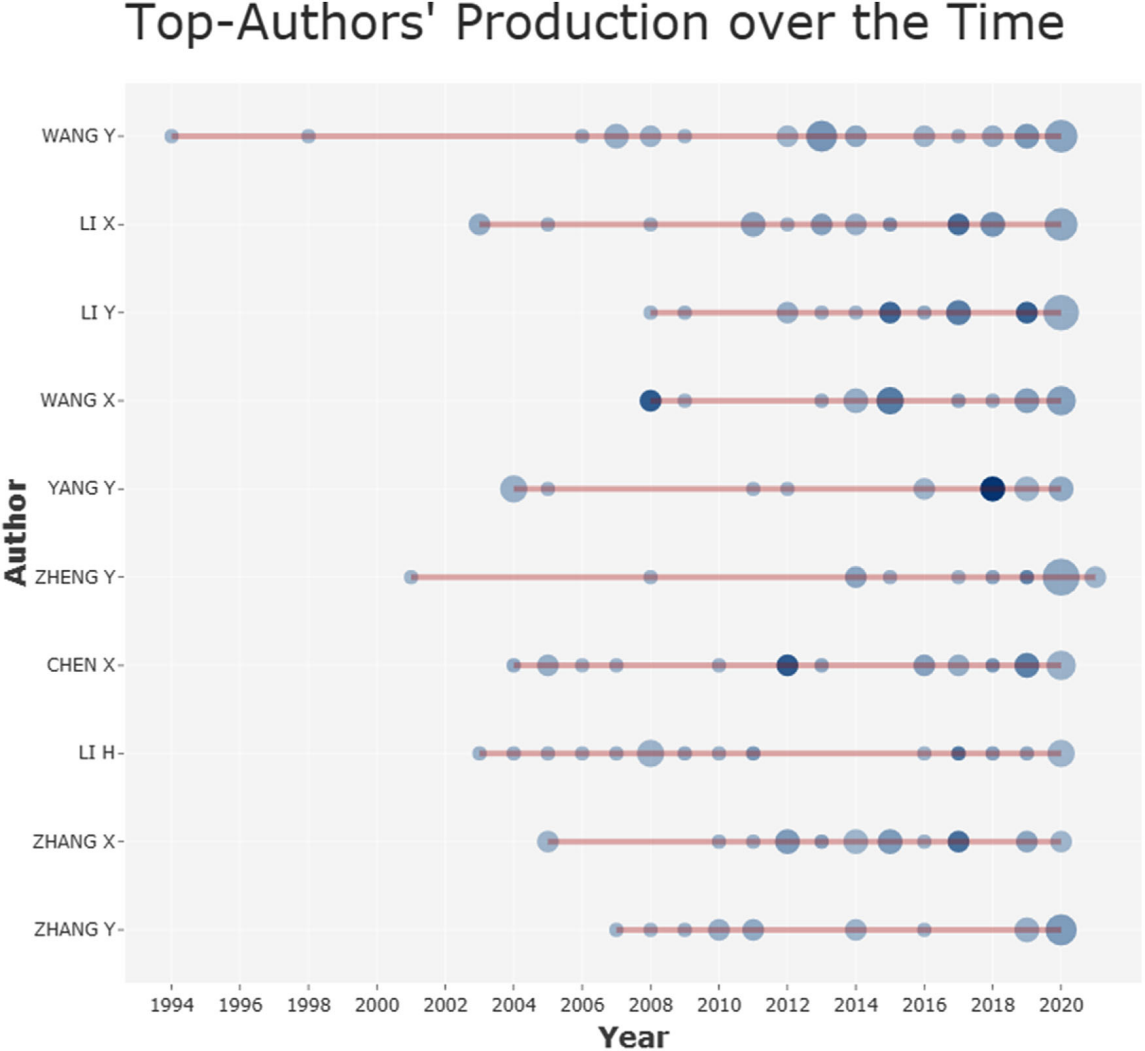
Top 10 most relevant authors’ production on medical image segmentation research field. (red line: publications start and end time, bubble size: the publications’ number,color intensity: number of citations of publications in that year)

In our analysis set of 1914 publications, there were a total of 4357 related authors appearing in the author list. Figure 7 shows the results of the top 10 authors with the largest number of publications. Among them, the most relevant author is Yu‐ping Wang from Tulane University, who published 31 articles in the medical image segmentation field. The red line in Figure 7 is the author's timeline, and the size of the bubble is in proportion to the number of publications that year, which means the larger the bubble is, the more publications related to the author in that year. The color intensity of bubbles is related to the total citations of publications in that year. The darker the color is, the higher the citation number of the author's publications in that year is. Figure 7 also illustrates that more and more researchers have joined the research field of medical image segmentation in the recent decade.

**FIGURE 8:**
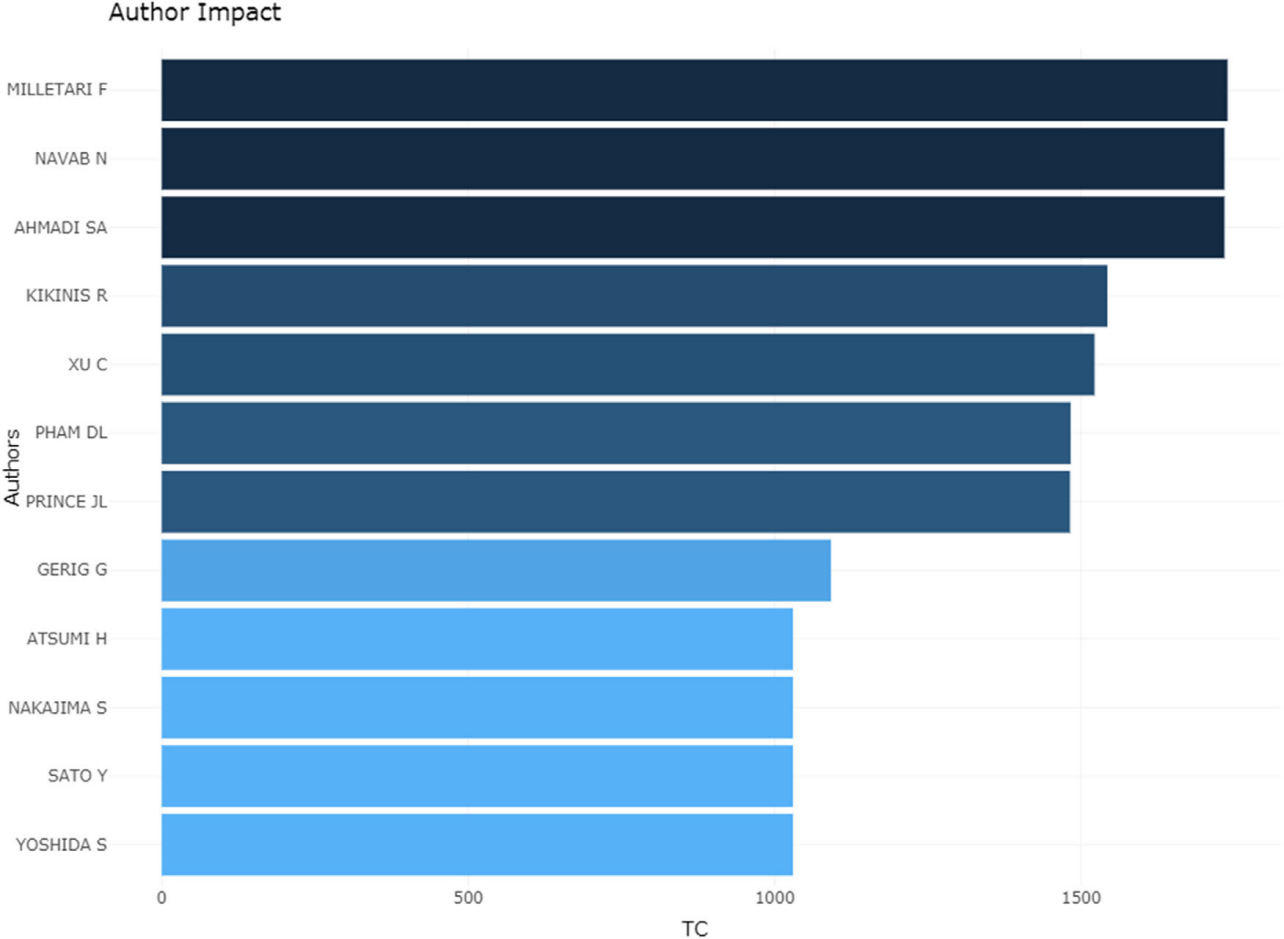
Top authors’ impact on the medical image segmentation research field

Figure 8 shows the authors, whose publications in the medical image segmentation field have been cited more than 1000 times, and illustrate the number of citations. A total of 12 researchers’ publications have been cited more than 1000 times. Among them, Fausto Milletarì from the Technical University of Munich ranked first, whose publications in this field had been cited 1739 times. Comparing Figures 7 and 8, we were surprised to find that most of the top authors are from China. However, none of these authors is a high‐impact author, which is also consistent with the results of the countries analysis in the previous section.

### 3.4 Sources analysis

**FIGURE 9:**
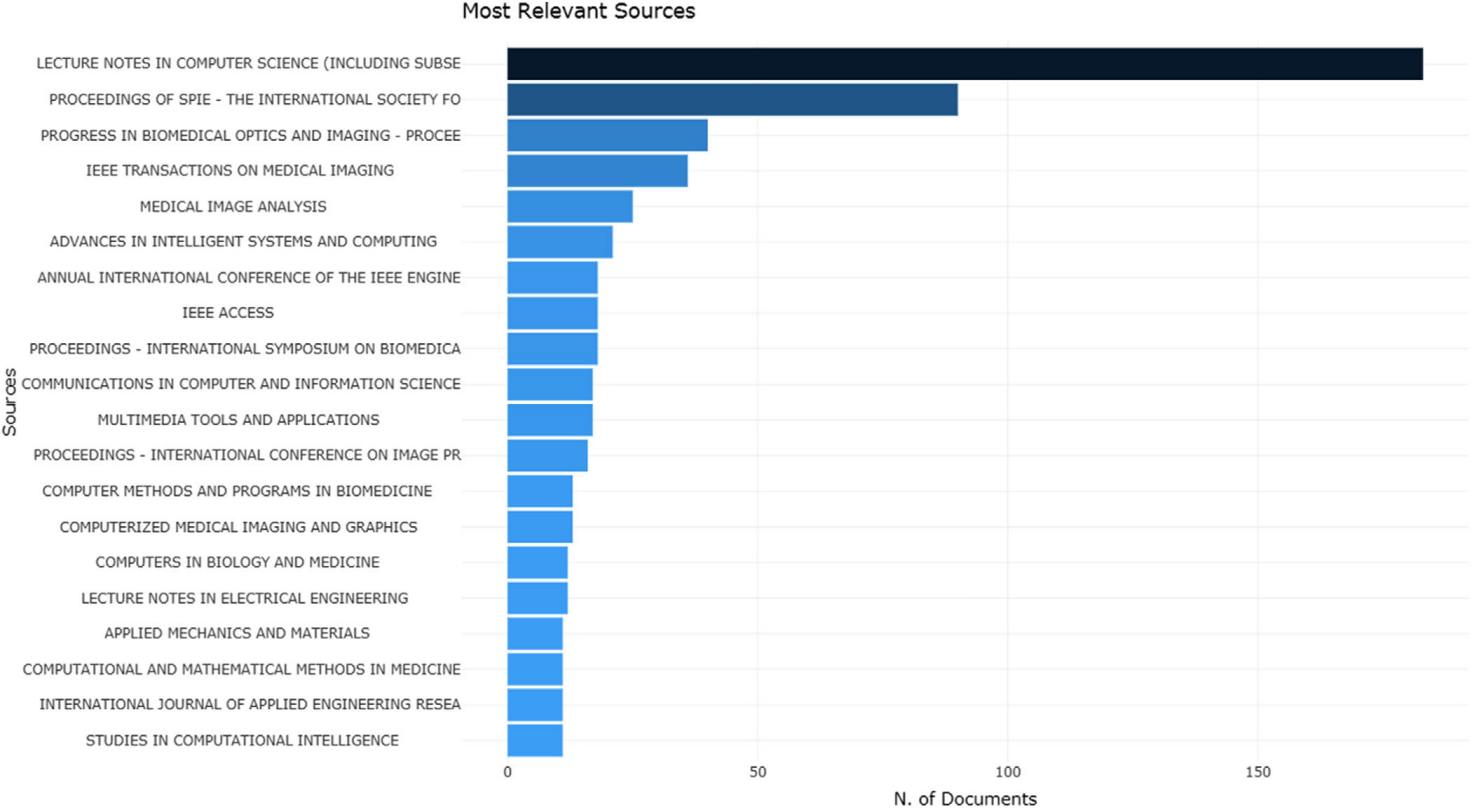
Top 20 sources that have the most number of publications on the medical image segmentation field

Figure 9 shows sources that have the most number of publications in the medical image segmentation research field. Among the top 10 publication sources, there are seven conference proceedings, and the top three are all conference proceedings. This is also consistent with the fact that more than half of the publications are conference papers. In terms of academic journals, “IEEE Transactions on Medical Imaging” is the journal with the largest number of publications in the medical image segmentation field, which published 36 academic articles. “Medical Image Analysis” is the second largest source journal, with 25 academic articles. Other top journals include 18 academic articles from “IEEE Access,”17 from “Multimedia Tools and Applications,” 13 from “Computerized Medical Imaging and Graphics,” 12 from “Computer Method and Programs in Biomedicine,” and 12 from ”Computers in Biology and Medicine.” These top journals are important for researchers in the medical image segmentation field when submitting research manuscripts.

### 3.5 Keywords analysis

Review articles collect information from tons of original research articles. The topic and keywords have lots of overlap. It affects the statistical data. Therefore, a total of 17 publications, which type was review, were removed from the analysis data set, and the keyword analysis was conducted on the remaining 1897 publications. Table 3 shows the most frequent author's keywords and their frequency of occurrence in medical image segmentation publications. The author's keywords that appeared at least 25 times in 1897 publications were listed. Among the 3042 author's keywords extracted from the dataset, only 24 have met the threshold. As the strongest three keywords, “image segmentation,” “medical image segmentation,” and “segmentation” represent the scope of research. The keywords related to image segmentation methods include “deep learning,” “level set,” “active contou*,” “fuzzy c‐means,” “region growing,” “clustering,” “deformable models,” and “edge detection,” which indicates the research hotspot and frontier of the medical image segmentation field.

**TABLE 3:** The most frequent author's keywords and their frequency of occurrence in medical image segmentation publications

Words	Occurrences
Image segmentation	342
Medical image segmentation	275
Segmentation	261
Medical image	112
Deep learning	77
Medical imaging	71
Medical images	51
Level set	51
MRI	43
Level set method	43
Active contour model	35
Region growing	33
Fuzzy c‐means	32
Roi	31
Clustering	31
Image processing	30
Active contour	29
Active contours	28
Fcm	28
Region of interest	28
u‐net	28
Deformable models	26
Edge detection	26
Intensity inhomogeneity	25

**FIGURE 10:**
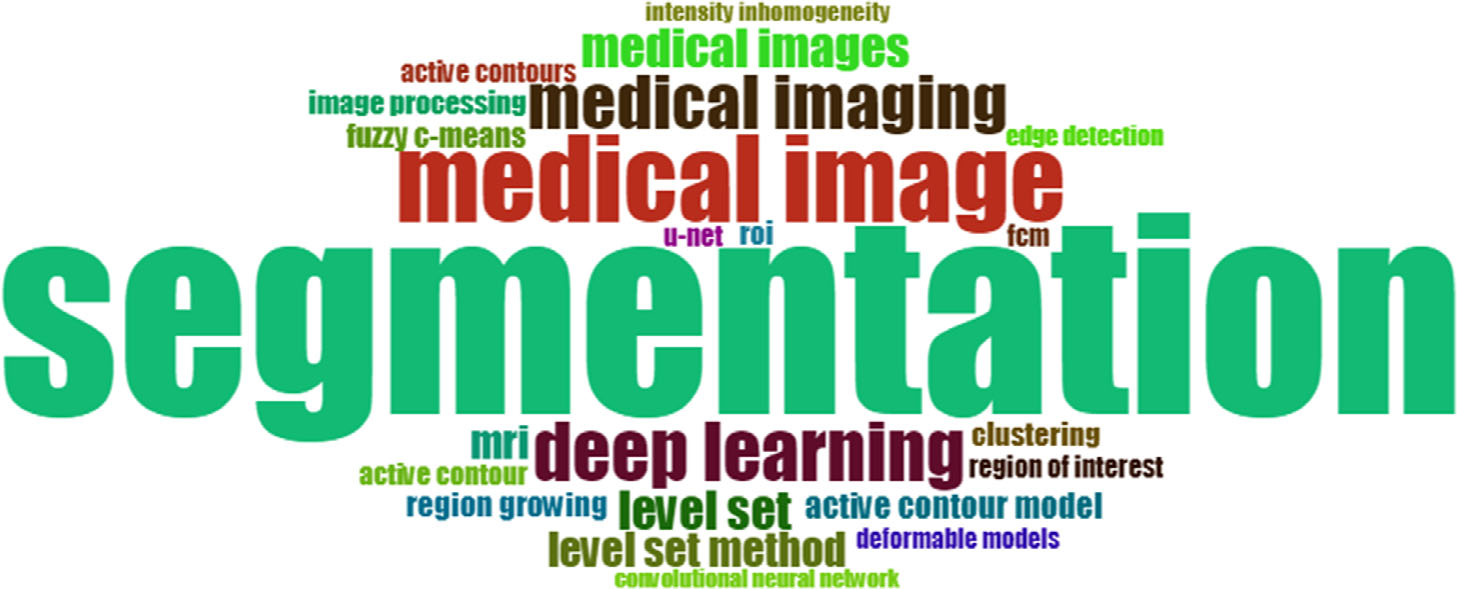
Word cloud of the most frequent author’s keywords in medical image segmentation publications. (font size: frequency of occurrence)

A Word cloud can visually illustrate the situation of keywords and highlight the keywords with a high frequency of occurrence. To grasp the most prominent keywords in the medical image segmentation field quickly and intuitively, word clouds are generated for the extracted author keywords. Figure 10 shows the word cloud of the author's keyword. The font size of a word or phrase represents its frequency of occurrence. It should be noted that Figure 10 and Table 3 have different types of forms, but their content is the same.

**FIGURE 11:**
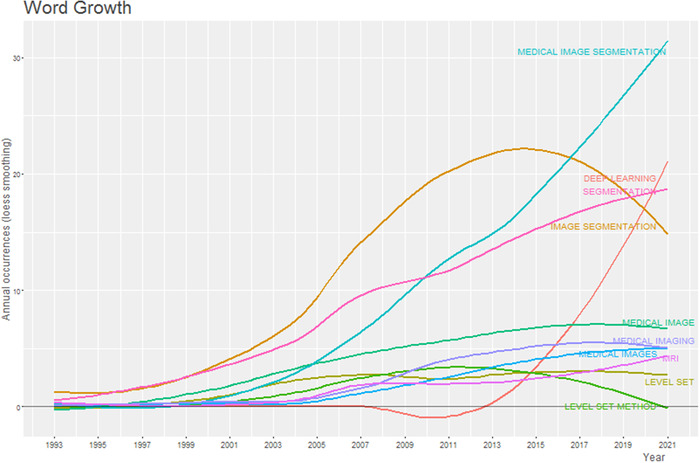
The variation trend (with Loess Smoothing) of 10 most frequent author’s keywords in medical image segmentation publications

Figure 11 shows the variation trend of the 10 most frequent author's keywords in the medical image segmentation field. The most obvious trend in the figure is that there are two curves, which represent “medical image segmentation” and “deep learning” respectively, growing rapidly in the past 10 years. This trend not only indicates that there are more and more publications in the medical image segmentation field, but also points out the research hotspot, which is deep learning‐based algorithms.

**FIGURE 12:**
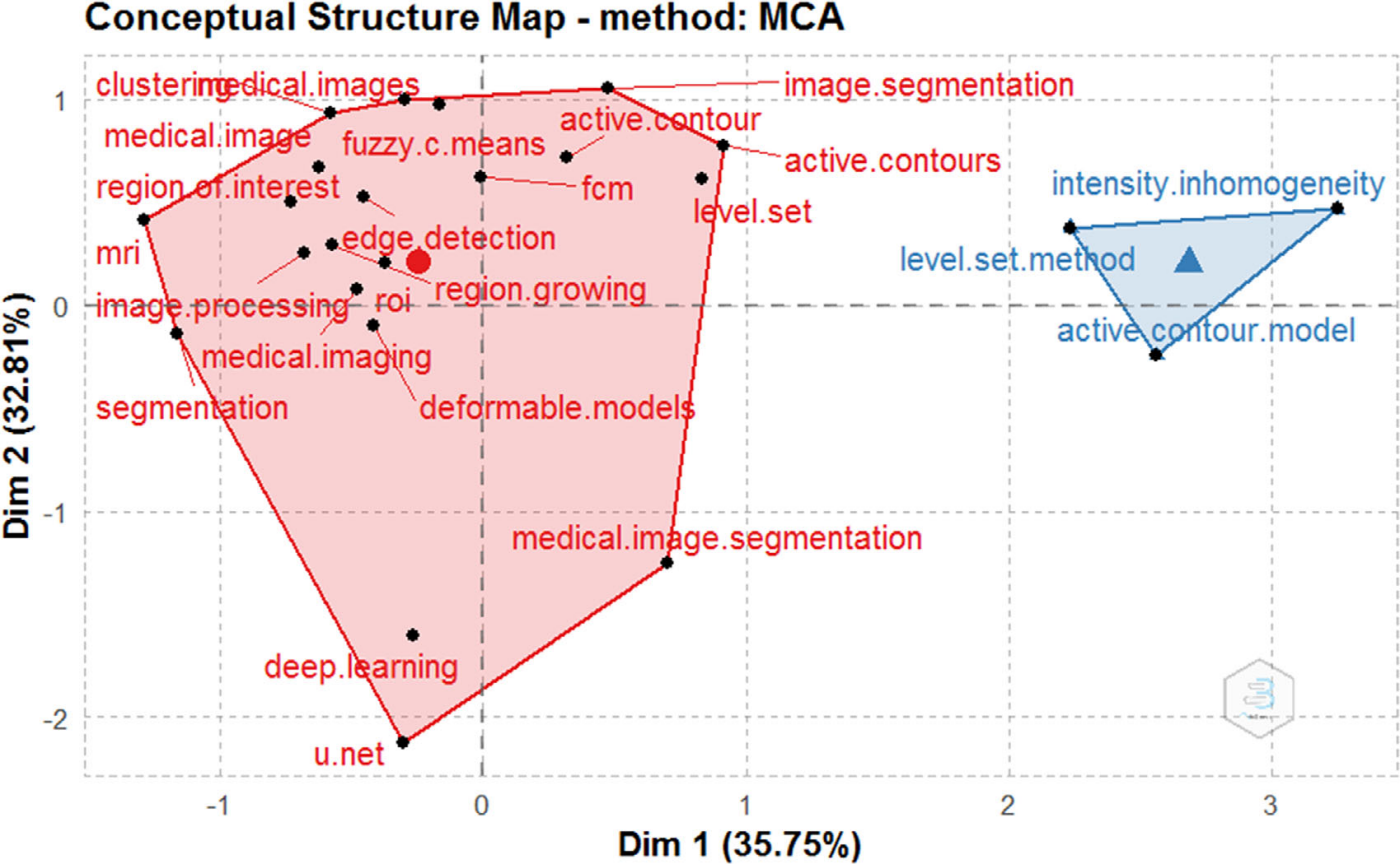
The Conceptual structure map of the most frequent author’s keywords in medical image segmentation publications

The most frequent author's keywords are listed in Table 3 and are divided into two categories by using the multidimensional scaling method, and the conceptual structure map was generated, as shown in Figure 12. The two dimensions of the map represent the average position of the publication included in each keyword, and the midpoint of the map represents the center of the medical image segmentation research field. It can be easily seen from the conceptual structure map that medical image segmentation publications can be divided into two main clusters. The red cluster contains most of the top keywords, while the blue cluster contains the remaining three keywords. Publications in these clusters contain detailed discussions of different types of methods for medical image segmentation.

**FIGURE 13:**
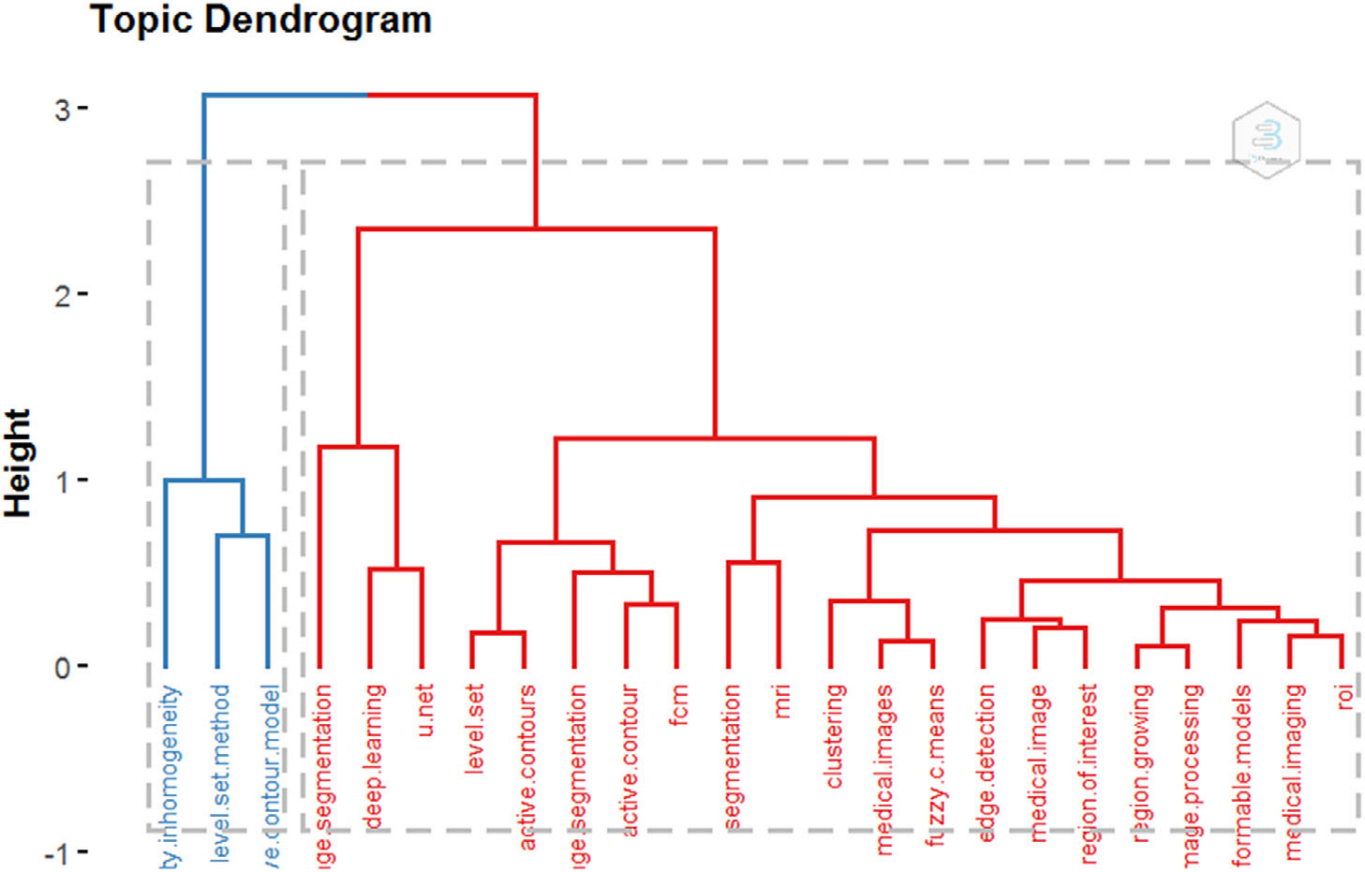
The topic dendrogram of the most frequent author’s keywords in medical image segmentation publications. (height: the distance between clusters or keywords)

Figure 13 is about the dendrogram of the medical image segmentation field, which is another form of the author's keywords conceptual structure map. At first glance, Figures 12 and 13 are different, but the information they contain is the same. Like the conceptual structure map, the dendrogram also divided the author's keywords into two clusters. The heights of the connection lines in Figure 13 have the same means as the distance between the keywords or clusters in Figure 12. Every color in the tree describes a partition, and the keywords connected by high connection lines often belong to different segmentation methods, which usually appear in different publications.

**FIGURE 14:**
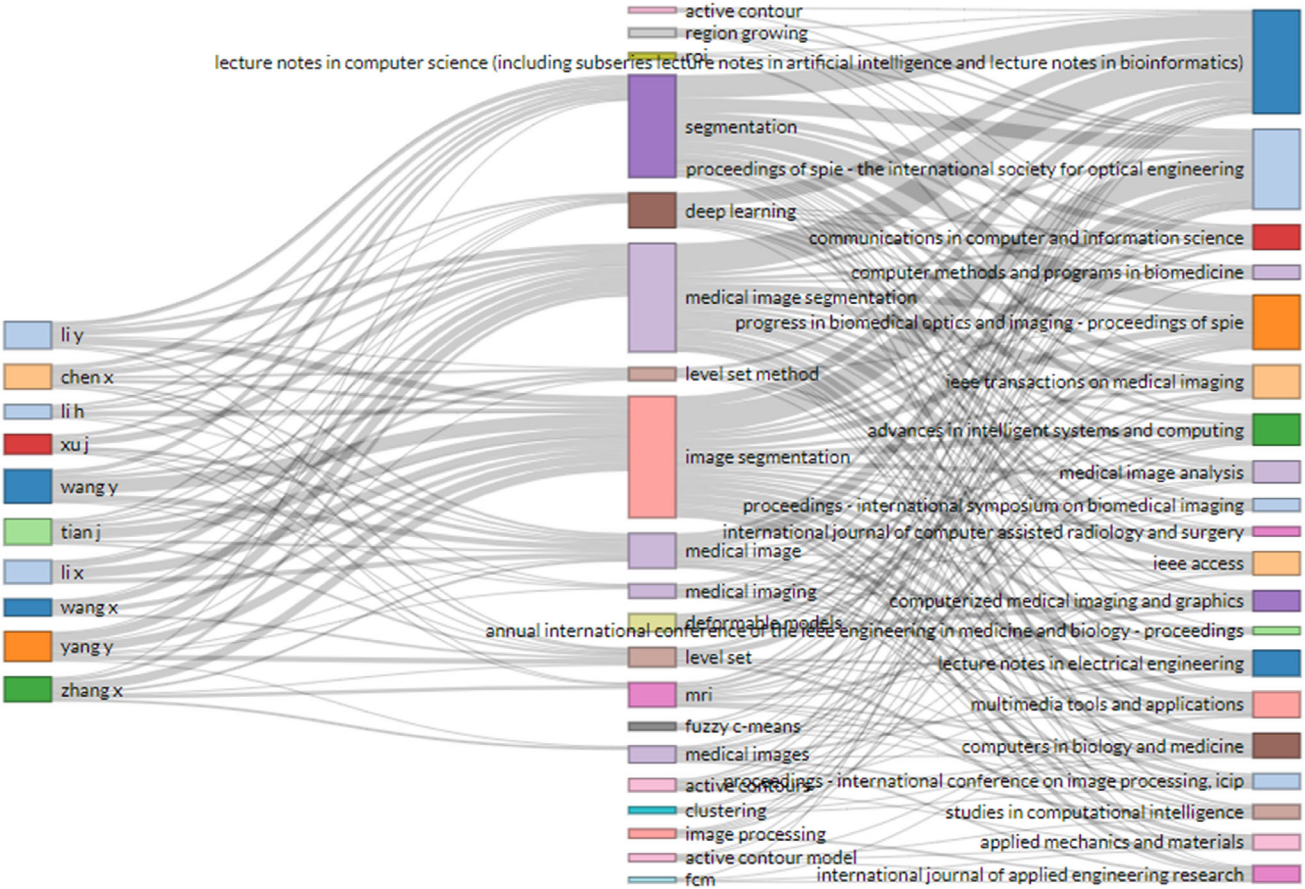
Three‐fields plot of the most frequent author’s keywords (the middle field), the top authors (the left field), and the top publication sources (the right field) and their relationships in medical image segmentation publications

Three‐field plots were used to analyze publications in the medical image segmentation field, which not only focuses on the most frequent author's keywords, the top authors, and the top publication sources but also illustrates the relationship among them, as shown in Figures 14. These three data sets were as the middle, left, and right fields of three‐field plots, respectively. While the height of each rectangle in the figure represents the number of keyword occurrences, the number of author's publications, or the number of publications from the source. As shown in Figure 14, Li Y, Li H, Wang X, Chen X, Xu J, Li X, Yang Y, Tian J, Wang Y, and Zhang X used almost all the top keywords in their publications. The most frequent author's keywords are “image segmentation,” “medical image segmentation,” “segmentation,” and “deep learning,” which are also the keywords of most of the top publication sources.

## 4 QUALITATIVE ANALYSIS

Up to January 11th, 2021, there were a total of 1914 publications in the medical image segmentation field in the Scopus database. To filter the most valuable high‐quality publications from this data set, all of them were sorted in descending order of annual citation rates, which is defined as the total number of citations divided by the number of years after publication. The publications whose annual citation rates were no less than 10 were filtered, and the results number was 49, accounting for 2.6% of the total number of publications. The annual citation rates of these publications ranged from 289.0 to 10.0. Qualitative analysis of the content of these top annual citation rates publications can help researchers to identify the most concerning research topics in the medical image segmentation field.

**FIGURE 15:**
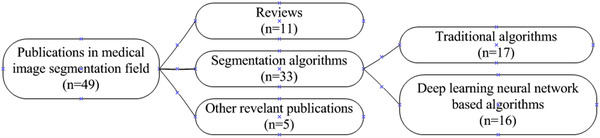
Taxonomy of top 49 annual citation rates publications in the medical image segmentation field

The taxonomy method was applied to classify these 49 high annual citation rates publications into three categories and several subcategories, as shown in Figure 15. The first category is review. The second category is medical image segmentation algorithms, which include two subcategories, the traditional segmentation algorithms, and the emerging deep learning neural network‐based segmentation algorithms. The third category is other relevant research, including medical image segmentation software and hardware design. These categories’ details are described in sections **4.1**‐**4.3**. These 49 publications with the top annual citation rates and their categories are listed in the Appendix section Table 4 in descending order of annual citation rates.

**TABLE 4:** TOP 49 annual citation rates publications and their categories. 1 (review), 2(1)(traditional segmentation algorithms), 2(2) (deep learning neural network based segmentation algorithms), and 3 (other relevant publications)

Rank	Annual citation rates	Title	Category
1	289.00	“V‐Net: Fully Convolutional Neural Networks for Volumetric Medical Image Segmentation^48^”	2(2)
2	68.75	“UNet++ A Nested U‐Net Architecture for Medical Image Segmentation^52^”	2(2)
3	67.92	“Statistical shape models for 3D medical image segmentation: A review^15^”	1
4	66.32	“Current methods in medical image segmentation^1^”	1
5	63.57	“Metrics for evaluating 3D medical image segmentation: Analysis, selection, and tool^65^”	3
6	43.24	“Interactive Medical Image Segmentation Using Deep Learning With Image‐Specific Fine Tuning^53^”	2(2)
7	40.20	“3D deeply supervised network for automated segmentation of volumetric medical images^51^”	2(2)
8	37.33	“CE‐Net: Context Encoder Network for 2D Medical Image Segmentation^59^”	2(2)
9	36.00	“Deep Learning Techniques for Medical Image Segmentation: Achievements and Challenges^31^”	1
10	35.46	“Three‐dimensional multi‐scale line filter for segmentation and visualization of curvilinear structures in medical images^34^”	2(1)
11	34.79	“A Shape‐Based Approach to the Segmentation of Medical Imagery Using Level Sets^44^”	2(1)
12	32.00	“SegAN: Adversarial Network with Multi‐scale L1 Loss for Medical Image Segmentation^55^”	2(2)
13	29.58	“Automated medical image segmentation techniques^16^”	1
14	28.22	“Improved Watershed Transform for Medical Image Segmentation Using Prior Information^36^”	2(1)
15	27.82	“Integrating spatial fuzzy clustering with level set methods for automated medical image segmentation^45^”	2(1)
16	23.75	“Learning Normalized Inputs for Iterative Estimation in Medical Image Segmentation^50^”	2(2)
17	23.61	“A novel kernelized fuzzy C‐means algorithm with application in medical image segmentation^4^”	2(1)
18	21.57	“Medical image segmentation on GPUs – A comprehensive review^8^”	1
19	19.50	“A review of algorithms for medical image segmentation and their applications to the female pelvic cavity^17^”	1
20	17.50	“An application of cascaded 3D fully convolutional networks for medical image^58^”	2(2)
21	17.11	“Medical Image Segmentation by Combining Graph Cuts and Oriented Active Appearance Models^38^”	2(1)
22	16.92	“A Geometric Snake Model for Segmentation of Medical Imagery^33^”	2(1)
23	16.88	“Medical Image Segmentation Methods, Algorithms, and Applications^29^”	1
24	16.67	“Active contour model based on local and global intensity information for medical image segmentation^9^”	2(1)
25	15.80	“Deep Learning for Multi‐task Medical Image Segmentation in Multiple Modalities^49^”	2(2)
26	15.67	“A novel segmentation model for medical images with intensity inhomogeneity based on adaptive perturbation^43^”	2(1)
27	15.67	“Data Augmentation Using Learned Transformations for One‐Shot Medical Image Segmentation^61^”	2(2)
28	15.50	“Convolutional neural network for bio‐medical image segmentation with hardware acceleration^67^”	3
29	15.29	“A comparative study of deformable contour methods on medical image segmentation^30^”	1
30	15.00	“Weighted Level Set Evolution Based on Local Edge Features for Medical Image Segmentation^47^”	2(1)
31	14.00	“DeepIGeoS: A Deep Interactive Geodesic Framework for Medical Image Segmentation^54^”	2(2)
32	13.75	“Segmentation of Dental X‐ray Images in Medical Imaging using Neutrosophic Orthogonal Matrices^41^”	2(1)
33	13.67	“Aleatoric uncertainty estimation with test‐time augmentation for medical image segmentation with convolutional neural networks^68^”	3
34	13.67	“NAS‐Unet: Neural Architecture Search for Medical Image Segmentation^60^”	2(2)
35	13.19	“Medical Image Segmentation Using K‐Means Clustering and Improved Watershed Algorithm^37^”	2(1)
36	12.86	“Dynamic‐context cooperative quantum‐behaved particle swarm optimization based on multilevel thresholding applied to medical image segmentation^40^”	2(1)
37	12.22	“Fast segmentation and high‐quality three‐dimensional volume mesh creation from medical images for diffuse optical tomography^39^”	2(1)
38	12.00	“Recurrent residual U‐Net for medical image segmentation^62^”	2(2)
39	11.90	“Interaction in the segmentation of medical images: A survey^28^”	1
40	11.75	“DRINet for Medical Image Segmentation^56^”	2(2)
41	11.71	“Medical Image Segmentation Using New Hybrid Level‐Set Method^46^”	2(1)
42	11.16	“Deformable M‐Reps for 3D Medical Image Segmentation^35^”	2(1)
43	11.00	“ASDNet: Attention based semi‐supervised deep networks for medical image segmentation^57^”	2(2)
44	11.00	“High‐resolution encoder–decoder networks for low‐contrast medical image segmentation^63^”	2(2)
45	10.85	“Medical Image Segmentation Using Genetic Algorithms^19^”	1
46	10.50	“Embracing imperfect datasets: A review of deep learning solutions for medical image segmentation^32^”	1
47	10.20	“A multi‐scale 3D Otsu thresholding algorithm for medical image segmentation^42^”	2(1)
48	10.00	“Accelerating compute intensive medical imaging segmentation algorithms using hybrid CPU‐GPU implementations^66^”	3
49	10.00	“A software tool for automatic classification and segmentation of 2D/3D medical images^2^”	3

### 4.1 Reviews analysis

There were 11 reviews among these 49 high annual citation rates publications in the medical image segmentation field, which occupied 22.4% of all high annual citation rates publications. This proportion is significantly higher than that of all reviews in the total publications, which was less than 1%. While the total number of reviews was 17, which indicated that more than 60% of these reviews on medical image segmentation had high annual citation rates. Therefore, reviews are classified into the first and most important category in this taxonomy. Qualitative analysis of these high annual citation rates reviews, which were published in different periods according to the time sequence, can help researchers to extract the most important information from the massive medical image segmentation publications and to grasp the research trend in this field from a macro perspective. In this subsection, these high annual citation rates reviews in the medical image segmentation field were analyzed according to the time of publication.

The research of medical image segmentation was started in the 1990s, and it was not until 2000 that a high annual citation rates review in this field was published, while the peak of review publication appeared around 2010. In 2000, Pham et al. reviewed medical image segmentation methods and divided them into eight categories, which are “thresholding, regional growth, classifiers, clustering, Markov random field (MRF) models, artificial neural networks, deformable models, and atlas guided approaches.^1^” Then the advantages and disadvantages of these types of methods were discussed. In 2001, Olabarriaga et al. surveyed interaction in the segmentation of medical images.^28^ The purpose of the study for interaction was limited by the research level in that year, sometimes automatic methods can fail and produce incorrect results, so manual intervention is often required for segmentation results.

With the prosperity of medical image segmentation research and a large number of research results published in this century, the number of reviews of medical image segmentation has increased gradually. In 2010, under the background of CT and MRI, Sharma and Aggarwal discussed automated medical image segmentation techniques and ranked them according to the applicability, suitability, performance, and computational cost, which are gray level and region‐based techniques, textural features‐based techniques, and neural network‐based algorithms.^16^ In another work, Ma, Z. et al. reviewed the medical image segmentation algorithms and their applications to the female pelvic cavity.^17^ These algorithms were discussed and classified into three categories, which are “thresholds based algorithms, clustering‐based algorithms, and deformable models‐based algorithms.” In 2014, Norouzi et al. discussed the advantages and disadvantages of four kinds of algorithms.^29^ These four kinds of algorithms are “region‐based methods, which include thresholding and region growing; classification methods, which include *k*‐nearest neighbor(k‐nn) and maximum likelihood; clustering methods, which include *k*‐means, Fuzzy C‐mean, and expectation maximization; and hybrid methods, graph cut (GCs).”

During this period, there are also some reviews of specific kinds of medical image segmentation methods that emerged. In 2008, He et al. conducted a comparative study on the application of eight different snake deformable contour methods and level set methods in medical image segmentation.^30^ In 2009, Tobias Heimann and Hans‐Peter Meinzer Div. reviewed the research progress of the statistical shape model in 3D medical image segmentation.^15^ In the same year, the genetic‐based medical image segmentation techniques were also reviewed.^19^ These genetic algorithms were divided into five categories by Ujjwal, namely “contour‐based technique, texture‐based technique, knowledge‐based technique, learning‐based technique, and model‐based technique.” In 2014, Smistad et al. discussed the most common medical image segmentation algorithms, evaluated their fitness to run on GPUs, and discussed several GPU optimization technologies.^8^

With the boom of artificial intelligence, deep learning, and neural network technology research in recent years, more and more deep learning and neural network‐based medical image segmentation research results have been published. In 2019, based on summarizing these works, Hesamian et al. reviewed the main training techniques of medical image segmentation neural networks and analyzed the advantages and disadvantages of them.^31^ In addition, the challenges of deep learning‐based medical image segmentation techniques were also discussed. In 2020, by summarizing the existing deep learning neural network‐based medical image segmentation methods, two problems of these algorithms are proposed, which are called “Scarce Annotations” and “Weak Annotations,” respectively.^32^ They pointed out that the reason for these problems is that training neural networks to segment medical images requires massive medical image data sets that are accurately segmented by human experts in advance, and the lack of these training data sets and error segmentation leads to the difficulties faced by deep learning neural networks‐ based medical image segmentation algorithms.

### 4.2 Segmentation algorithms analysis

The high annual citation rates publications on medical image segmentation algorithms are reviewed in this subsection. These publications are further classified according to different types of algorithms including traditional medical image segmentation algorithms, and deep learning neural network‐based medical image segmentation algorithms. There are a total of 33 publications in this category, accounting for 67.3% of the total number of high annual citation rates publications, covering a period from 1994 up to now. Qualitative analysis of these publications can help researchers to delve into the research details in this field on a microlevel.

#### 4.2.1 Traditional algorithms

There were only two publications in the last century on traditional medical image segmentation algorithms with high annual citation rates including the geometric snake model^33^ and the three‐dimensional (3D) multiscale line filter.^34^ In the first decade of the 21st century, more and more high annual citation rates publications on traditional medical image segmentation algorithms emerged, which include deformable M‐Reps,^35^ fuzzy C‐means,^4^ and two different improved watershed transform‐based algorithms.^36^, ^37^

In the recent decade, although the research hotspot and frontier of medical image segmentation have changed to deep learning neural network‐based algorithms, there are still some traditional medical image segmentation algorithms that have been proposed. In 2012, Chen et al. proposed a 3D abdominal 3D organ segmentation method based on the combination of active appearance model (AAM), live wire (LW), and GCs.^38^ In 2013, Jermyn et al. proposed a diffuse optical tomography‐based medical image segmentation method.^39^ In 2014, Y. Li et al. proposed a “multilevel thresholding‐based dynamic‐context cooperative quantum‐behaved particle swarm optimization,” which can be applied to medical image segmentation.^40^ In 2017, Ali et al. proposed a fuzzy clustering and neutrosophic orthogonal matrices‐based medical image segmentation method and applied it to dental X‐ray images.^41^ In the same year, Zhang et al. proposed a multiscale 3D Otsu threshold segmentation scheme, which improved the accuracy of one‐dimensional Otsu segmentation.^42^ In 2018, Yu et al. proposed a “new edge‐based active contour model” for medical image segmentation.^43^ While among these high annual citation rates traditional algorithms, the level set method has been most applied.^9^, ^44^, ^45^, ^46^, ^47^

#### 4.2.2 Deep learning neural network‐based algorithms

In the recent 5 years, with the emergence of artificial intelligence technology, deep learning and convolutional neural network (CNN)‐based medical image segmentation algorithms have also been developed greatly. Although the high annual citation rates publications in this field only began in the last 5 years since 2016, the publication number was as high as 18, accounting for more than 50% of the publications on medical image segmentation algorithms, and the growth trend is accelerating. These publications were analyzed in chronological order in the following section.

In 2016, Milletari et al. proposed a “convolutional neural network (VNET)‐based volumetric medical image segmentation,” which can segment prostate tissue fastly and accurately in MRI images.^48^ In another work, Moeskops et al. proposed a deep learning and single CNN‐based multitask and multiple modalities medical image segmentation.^49^ There were only two high annual citation works published this year, but the literature^48^ has attracted a great attention in the medical image segmentation field. Since its publication, the article has been cited 1734 times and the annual citation rates were as high as 289.0, which was four times as high as that of the second most cited literature of the entire analysis set.

As well as 2016, there were two deep learning and CNN‐based medical image segmentation publications that received a high annual citation rates in the year 2017. Combining the fully convolutional networks (FCNs) and the fully convolutional residual networks (FC‐ResNets), Drozdzal et al. proposed a “simple and powerful pipeline for medical image segmentation.^50^” A new 3D deeply supervised network was proposed by Dou et al., which can be applied to segment the liver from 3D CTs and the whole heart and great vessels from 3D MRIs.^51^

In 2018, the number of high annual citation rates publications for deep learning and CNN‐based medical image segmentation algorithms began to increase dramatically, and a total of seven publications entered the high annual citation rates list. Zongwei et al. proposed a nested U‐net architecture named Unet++ for medical image segmentation, which is a deeply supervised encoder‐decoder network in essence.^52^ Wang et al. proposed a CNNs‐ and pipeline‐based segmentation framework with user intervention which can also work without user intervention.^53^ Another outstanding advantage of the CNNs is that they can be used to segment untrained medical targets, and the segmentation accuracy is only slightly worse than that of trained targets. In another of his work, they proposed a deep interactive geodesic framework for medical image segmentation, named DeepIGeoS, which adopted the intervention of medical image experts to improve the accuracy of results obtained by automatic CNN.^54^ Inspired by the classic generative adversarial networks (GANs), Xue et al. proposed a novel end‐to‐end adversarial neural network, called SegAN, for medical image segmentation.^55^ This network is a multiscale loss framework, and is very effective and leads to more superior performance when compared with single‐scale loss or conventional pixel‐wise softmax loss. Chen et al. proposed a new CNN structure, named Dense‐Res‐Inception Net (DRINet), which is used for medical image segmentation.^56^ To improve the problem that neural network methods have a high dependence on the accuracy of training data annotation, Nie et al. proposed a semisupervised network that combined image segmentation network with confidence network.^57^ As for hardware‐related CNN‐ based medical image segmentation, Roth et al. proposed a cascaded 3D fully convolutional networks (3D U‐NET) and deployed it on a single GPU.^58^

In 2019, there was some reduction of high annual citation rates publications for deep learning and CNN‐based medical image segmentation algorithms, and a total of five articles entered the high annual citation rates list this year. Gu et al. proposed a “context encoder network for 2D medical image segmentation,” named CE‐Net, which was applied to several different tasks.^59^ Weng et al. proposed a neural architecture search for medical image segmentation, named NAS‐Unet, which has better performances and fewer parameters than U‐net when processing MRI, CT, and ultrasonic images.^60^ Zhao et al. presented a learning‐based method for data augmentation and demonstrated it on one‐shot medical image segmentation.^61^ Based on the U‐Net model, Alom et al. proposed a recurrent U‐Net model and a recurrent residual U‐Net model, which are named RU‐Net and R2U‐Net, respectively.^62^ Three different benchmark datasets were used to test the proposed models including retinal vascular segmentation, skin cancer lesion segmentation, and LS. The result showed that it has good performance when compared with SegNet, U‐Net, and residual U‐Net. To solve the problem of fuzzy boundary detection in the existing neural network‐based medical image segmentation method, Zhou et al. proposed a neural network containing three pathways, which are Distilling Pathway, High‐resolution Pathway, and Contour Information Integration.^63^

In 2020, there is not any publication received more than 10 times citation. This may be due to the fact that the manuscripts that cited recent publications have not yet been published. Manuscripts usually take 3–6 months or more to be published.

#### 4.2.3 Segmentation accuracy analysis of deep learning neural network‐based algorithm

Segmentation accuracy is the most important index to evaluate a medical image segmentation algorithm and should be the primary concern of relevant researchers. The analysis in Section**4.2.2** reveals the boom of deep learning neural networks in the medical image segmentation field in recent years. To further compare these algorithms, in this section, algorithms mentioned in Section 4.2.2 are compared under dice value. The dice value is defined by Equation (1).

(1) $$Dice=\frac{2(A\cap B)}{A+B}$$

*Here*

*A and B represent the predicted region and the ground truth region, respectively*.

The dice value is between 0 and 1. The value is closer to 1, the segmentation accuracy is higher. It should be pointed out that, under the same algorithm, the dice values of different types of medical images or different tasks are often quite different. Although it is meaningful to compare the dice values given directly in these publications, it is more meaningful to analyze the differences under the same segmentation task. Among them, the U‐net^64^ and FCN^48^ are often used for horizontal comparison. As shown in Table 5, all the algorithms have achieved high segmentation accuracy relatively, but there is still some space for improvement.

**TABLE 5:** The comparison among the 16 deep learning neural network‐based medical image segmentation algorithms in the aspect of Segmentation accuracy (Dice value)

Publication title	Segmentation accuracy (Dice value)(%)	Dice value comparison in the same task
U‐Net: Convolutional networks for biomedical image segmentation^64^	84.80	U‐net
V‐Net: Fully Convolutional Neural Networks for Volumetric Medical Image Segmentation^48^	86.90	FCN
Deep Learning for Multi‐task Medical Image Segmentation in Multiple Modalities^49^	81	
Learning Normalized Inputs for Iterative Estimation in Medical Image Segmentation^50^	87.40	
3D deeply supervised network for automated segmentation of volumetric medical images^51^	92.80	1.8% higher than U‐net
UNet++ A Nested U‐Net Architecture for Medical Image Segmentation^52^	70.56	3.32% higher than U‐net
Interactive Medical Image Segmentation Using Deep Learning With Image‐Specific Fine Tuning^53^	87.12	1.84% higher than FCN 3.55% higher than U‐net
DeepIGeoS: A Deep Interactive Geodesic Framework for Medical Image Segmentation^54^	87.62	4.39% higher than FCN
SegAN : Adversarial Network with Multi‐scale L1 Loss for Medical Image Segmentation^55^	85	
DRINet for Medical Image Segmentation^56^	91.85	0.94% higher than FCN 0.82% higher than U‐net
ASDNet: Attention based semi‐supervised deep networks for medical image segmentation^57^	92.90	8.2% higher than FCN 8.7% higher than U‐net
An application of cascaded 3D fully convolutional networks for medical image^58^	90.13	4.3% higher than FCN
CE‐Net: Context Encoder Network for 2D Medical Image Segmentation^59^	96.20	4.9% higher than U‐net
NAS‐Unet: Neural Architecture Search for Medical Image Segmentation^60^	98.50	0.7% higher than U‐net
Data Augmentation Using Learned Transformations for One‐Shot Medical Image Segmentation^61^	80.40	
Recurrent residual U‐Net for medical image segmentation^62^	96.50%	0.9% higher than U‐net
High‐resolution encoder–decoder networks for low‐contrast medical image segmentation^63^	90.10%	1.67% higher than FCN

### 4.3 Other relevant publications analysis

Apart from the categories identified in sections 4.1 and 4.2, there are some other high annual citation rates publications in the medical image segmentation field which are combined in this section. In 2013, Strzelecki et al. designed a software package named MaZda, which supports the analysis, classification, and segmentation of 2D/3D medical images.^2^ In 2015, Taha et al. developed metrics for evaluating 3D medical image segmentation.^65^ These metrics use 20 evaluation indicators and are open source for researchers. In 2016, Alsmirat et al. used the GPU to accelerate the processing time of medical image segmentation.^66^ Without reducing the segmentation accuracy, the segmentation speed is 8.9 times faster than using the CPU alone. In 2018  Vardhana et al. proposed a low power detection system for edge detection of medical images, in which a CNN was used for image classification and disease diagnosis, and hardware was used to accelerate the processing.^67^ In 2019, Wang et al. proposed an uncertainty estimation method and used it to analyze the uncertainty of CNN‐based medical image segmentation algorithms of different dimensions at different segmentation levels.^68^

## 5 LIMITATIONS

This bibliometric is first limited by the number of academic databases searched, especially not including non‐English databases, although the selected databases are reliable and comprise broadly representative collections. Second, the rapid progress in the field of medical image segmentation technology limits the timeliness of the bibliometric. Third, we emphasize that research activities in this field do not necessarily reflect the practical application or impact of these research findings in this field. The results of these studies only reflect current research trends in academia, which is the purpose of this bibliometric.

## 6 CONCLUSIONS

In this work, quantitative and qualitative analysis was conducted on publications in the medical image segmentation field get from the Scopus database as of January 11th, 2021. In the quantitative analysis stage, a total of 1914 publications were analyzed from five perspectives: annual publications, countries, top authors, publication sources, and keywords. The results showed that the number of publications in the medical image segmentation field increased rapidly by year, and the citation rate also increased as a whole. Most of the authors were from the Chinese mainland, the United States, and India, and there was a lot of cooperation between the Chinese mainland and the United States. Most of the top 10 most relevant authors were from the Chinese mainland, but none of them were high‐impact authors. More than half of the 1914 publications were conference papers, and “IEEE Transactions on Medical Imaging,” “Medical Image Analysis,” and “IEEE Access” were the top three journals. The keywords analysis showed that deep learning is the research hotspot in this field in recent years. In the qualitative analysis stage, 49 publications with no less than 10 annual citation rates were analyzed. The taxonomy method was applied to classify these publications into three categories including reviews, segmentation algorithms, and other relevant publications. Among these three categories, segmentation algorithms accounted for the vast majority, among which deep learning neural networks‐based algorithms were the research hotspots and frontiers. This article has a significant meaning for understanding medical image segmentation and following the direction of further research in this field. It can be expected that more and more researchers will work on the deep learning neural network‐based medical image segmentation, which may lead to more and more publications.

## AUTHORS’ CONTRIBUTIONS

**Bin Zhang**: Literature search and data analysis, writing original draft preparation, and obtain funding support.

**Bahbibi Rahmatullah**: Supervision and revised the manuscript.

**Shir Li Wang**: Supervision and revised the manuscript.

**Guangnan Zhang**: Writing review and editing, obtain funding support.

**Huan Wang**: Writing review and editing, obtain funding support.

**Nader Ale Ebrahim**: provides the methodology and revised the manuscript.

## CONFLICT OF INTEREST

The authors declare that they have no conflict of interest.
